# Paraffin wax as self-sealing insulation material of seasonal sensible heat storage systems—A laboratory study

**DOI:** 10.1371/journal.pone.0236056

**Published:** 2020-07-29

**Authors:** Christoph Bott, Ingo Dressel, Peter Bayer

**Affiliations:** 1 Institute of New Energy Systems, Ingolstadt University of Applied Sciences, Ingolstadt, Germany; 2 Institute of Geosciences and Geography, Martin Luther University Halle-Wittenberg, Halle, Germany; 3 K+S AG, Kassel, Germany; Texas A&M Univ, UNITED STATES

## Abstract

Seasonal heat storage is considered as one of the key elements on the path to a low-emission economy. Embedded in local district heating networks, they raise the share of renewable energies and balance out highly fluctuating supplies of e.g. solar systems or windmills. The technology of seasonal heat storage can be described as almost technically mature, with well-established concepts and some systems being in operation for a considerable time. Nevertheless, the operating experience gained to date also revealed two critical problems. On the one hand, even smallest leakages in sealing foils led to irreparable breakdowns. On the other hand, heat loss in the marginal areas was revealed as a key deficiency, preventing the technology from advancing towards global marketability. This study presents an experimental approach to address these two key issues in the field of seasonal energy storage. Two small-scale laboratory tests were carried out to test paraffin wax as a completely novel component in the marginal area of seasonal storages. This is based on two material properties: As hydrophobic and mobile medium, the warmed and molten paraffin should actively seal the fissures and holes in the event of leakage. Additionally, the latent heat storage properties of the paraffin wax should increase the systems’ total storage capacity and reduce lateral heat losses via its low thermal conductivity. With retardation periods from 2.5 to 4 hours, the results show an effective phase change effect of the paraffin wax, which reduces energy losses and allows to buffer short-term, intensive loading and unloading processes. By storing up to 138 kJ/kg energy in the paraffin wax, increased capacities of application-scale pit storages by up to 40.70 MWh are to be expected. Additionally, the self-healing features could be successfully demonstrated: With only small losses of between 1.5 and 17%, the paraffin wax effectively sealed artificially incised leaks. Thereby, the mechanism was most effective for local defects. Following these positive demonstrations of feasibility, technical design questions still remain, which concern prevention of deformation of the paraffin wax. Once solved, this new component can then provide a path for further optimization of seasonal heat storage technologies.

## 1 Introduction

Seasonal heat storage has evolved as a promising strategy for storing thermal energy from fluctuating sources over long periods. Solar energy collected in the summer or any momentarily available excess heat can be stored for feeding a district heating network during the winter season [1–3]. The wide variety of available concepts covers latent, chemical and sensible variants [4–7]. While latent heat storages utilize phase change effects (e.g. of water/ice or hydrocarbons) [8–10], thermochemical storages are based on reversible endo- and exothermic reactions, such as salt hydrations [11, 12]. However, both of these concepts are often not applicable to large-scale applications due to high material costs. Sensible heat storage, in contrast, features the utilization of temperature changes [13–16]. In this context, large seasonal storage systems are generated via borehole fields (Borehole Thermal Energy Storage [17, 18]) or wells in aquifers (Aquifer Thermal Energy Storage, e.g. [19–21]). Another common technological variant, which is also the focus of this study, is the storage of thermal energy in large, artificial, ground-based basin structures. In these, water or water-filled gravel with volumes of several thousand cubic meters are used as storage media [14, 22–24]. Especially for Pit Thermal Energy Storages (PTES) and Water-Gravel Thermal Energy Storages (WGTES), standard solutions for thermal insulation are non-existent. However, long-term thermal storage efficiency strongly depends on a competent and reliable technique that minimizes lateral heat loss from the basin [2, 25]. This means, the storage media needs to be embedded in a stable waterproof shell of low thermal conductivity. This shell thus commonly consists of an internal impermeable sealing layer, that is, plastic foils encapsulating the water in the basin [23, 26–28]. For thermal insulation, highly porous and relatively cheap materials such as layers of foam glass or expanded glass gravels are used. As these are not resistant against the high structural loads of the overlying storage media, most of the previously constructed storage systems are insulated only at the top and side walls [1, 29, 30].

A main barrier for seasonal thermal storage basins to reach market maturity relates to technical vulnerabilities of the sealing and insulation components. The assessment of numerous existing sites reveals that there are two most important categories of deficiencies: 1) Efficiencies/heat losses: In many cases, higher energy losses and lower system efficiencies were measured than previously predicted (e.g. [29, 31, 32]). The reasons for heavily reduced performances were, among others, water ingress into the thermal insulation and interaction with groundwater, promoting the dissipation of heat [33–36]. Ultimately, these high energy losses reduce the efficiency of the storage facilities, raising questions about their general economic viability [37–39]. 2) Leakages: A significant number of systems suffered from leaks, leading to irreparable damages and sometimes even total failures (e.g. [34]). Insufficient long-term resistance of the materials used for basin sealings could not withstand the highly variable thermal conditions and the static load of the filling material. Material fatigue is further intensified by the contrasting cold environment and hot storage filling during intense charging and discharging processes [28, 33]. As large-scale storage basins thus dispense with the cost-intensive use of insulation, sealing layers are positioned directly on the surrounding soil and are exposed to a higher risk of injury. It is striking that most problems occur in the transition area from the storage media to the surrounding environment.

Based on these key issues, this study examines the suitability of a novel membrane concept based on paraffin wax, which may be suited for simultaneous insulation and sealing of seasonal heat storage basins. Paraffin wax is a mixture of hydrocarbon molecules with varying numbers of carbon atoms. The lengths of the C-chains range between 20 and 60 for soft and hard paraffin waxes and this controls both melting and solidification points of the bulk material used. For example, for a solidification point of 42 °C and a melting point of 40 °C, the molecules have a chain length of around 21 C-atoms. With an enthalpy of fusion between 150 kJ/kg and 220 kJ/kg, paraffin wax is one of the most popular storage materials [7, 40–45]. Its thermal conductivity is relatively low, with values of 0.15 W/m K to 0.30 W/m K around one order of magnitude below that of a water-saturated gravel (2.4 W/m K in the case of an implemented Water-Gravel Thermal Energy Storage described in [38]) [46, 47]. Aside from this, it is hydrophobic and non-toxic [48]. These favorable properties support the use of paraffin wax for lateral thermal insulation and energy absorption, while melting of paraffin wax consumes energy and thus keeps it in the system [43–45, 49]. A recuperation effect may be utilized when the storage cools down and paraffin wax solidification enables recovery of the heat stored in the phase change. The conventional use of paraffin wax as a thermal storage medium already makes use of these effects in various respects [50–53]. In encapsulated form or as a composite material with polymers, it has been employed, for example, for thermal component activation within buildings and for small-scale thermal storage applications [9, 54–56]. However, if the paraffin wax is integrated directly and made available as a mobile medium in its liquid, molten state, it may be able to clog leakages in the sealing layers of storage systems via its hydrophobic property. In direct contact with cold surroundings next to the insulation membrane, it would cool down and prevent water loss from the basin. The objective of this work is to investigate these expected self-sealing properties of paraffin wax considering the conditions of seasonal storage applications in downscaled laboratory experiments.

By integrating a latent heat storage material in the marginal section, the critical vulnerabilities of existing storage membrane concepts are attacked while, at the same time, new benefits are added. Thereby, the presented approach is both technically simple and somewhat paradoxical, as paraffin wax is already a well-established material for thermal energy storage. Thus, this study provides a substantially new application strategy for thermal insulation.

With the objective of providing an initial proof of feasibility and to demonstrate the applicability of the intended mechanisms, this study is divided into two separate sections. The first part inspects the thermal performance of paraffin wax in a multi-stage laboratory experiment. Here, variants of implementing paraffin wax as insulation material are tested at variable temperature ranges. The second part is dedicated to the imperviousness of the storage membrane. For this purpose, various types of artificially induced leakages and selected surrounding materials are analyzed and the paraffin wax’s migration behavior is scrutinized.

## 2 Materials and methods

### 2.1 Enhancement of thermal performance

#### 2.1.1 Experimental setup

**Fig 1:**
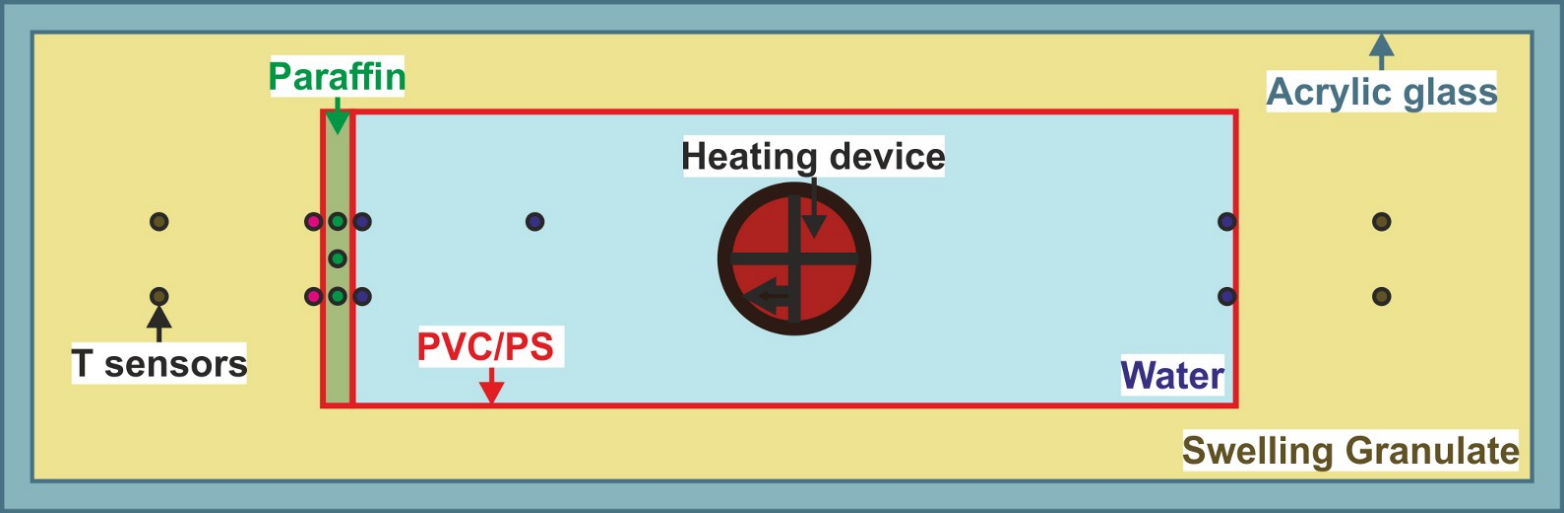
Schematic top view of the thermal performance experimental setup showing the positions of temperature sensors and materials used. PVC: polyvinylchloride foil, PS: polystyrene glass plates.

**Fig 2:**
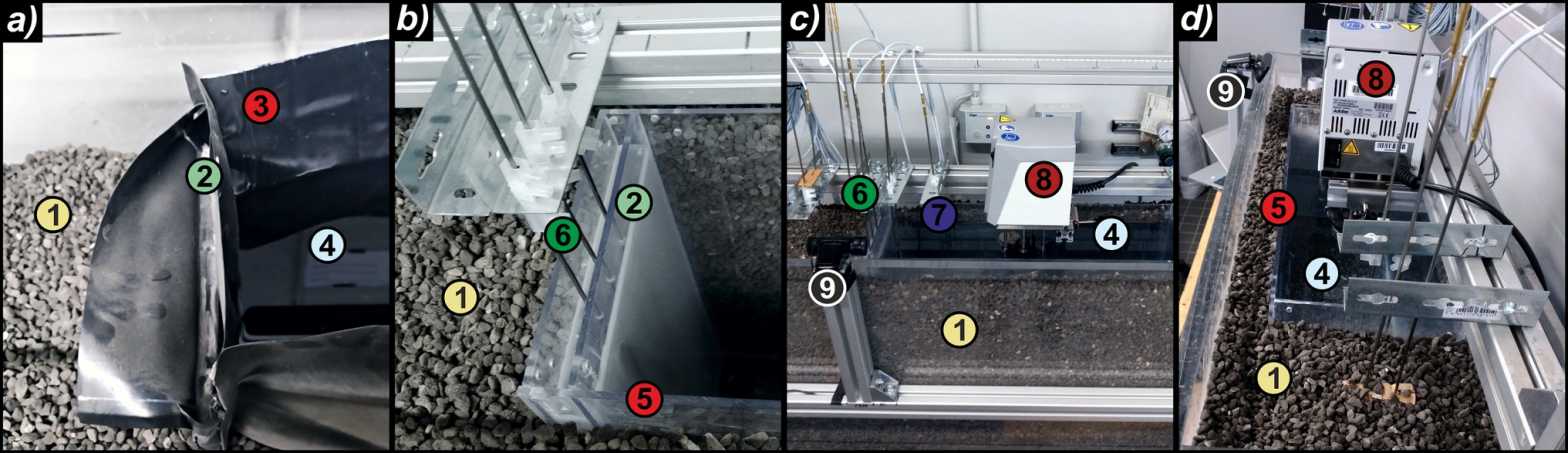
Experimental variants with (a) black PVC foil and (b-d) PS as sealing layer. 1: surrounding material, 2: paraffin wax insulation layer, 3: PVC sealing foil, 4: filling/water, 5: PS sealing plates, 6, 7: temperature sensors in paraffin wax/filling, 8: heating device, 9: camera.

The first laboratory test was designed to investigate energy losses when using paraffin wax inside two sections of sealing layers of a PTES structure. A schematic illustration of the experimental setup is given in Fig 1, while Fig 2 shows images of the erected set up in the laboratory.

As outer enclosure, a container made of acrylic glass with internal dimensions of 1000 mm x 300 mm x 600 mm (length, width, height) was used. A small-scale heat storage with deionized water as filling material was implemented inside, with its inner dimensions (600 mm x 200 mm x 400 mm) encapsulated by the inner sealing component. In the first series of experiments, sealing was conducted via rigid 5 mm thick plates of polystyrene glass (PS). In the second series, the PS plates were replaced by a 0.5 mm thick polyvinylchloride (PVC) foil, which is commonly used for sealing in existing storage basins [1, 26, 57]. Comparing the use of non-formable PS plates with flexible, standard PVC foils facilitates to focus on potential mechanical deformation when including the paraffin wax. The latter was cast between another layer of the sealing membrane on one short side of the container (Fig 2a and 2b). The form of unadulterated paraffin wax was chosen to exploit the direct availability as a molten liquid to reseal leakages in the second part of the experiments. Within the sealing membrane, the paraffin wax was distributed over the entire surface without pore spaces, which would not be the case with the paraffin composite materials frequently used in the construction sector, such as encapsulated paraffin wax. Here, this simultaneously provides a larger volume for additional storage of energy. In the case of the PS plates, a cavity distance of 20 mm was implemented (Fig 2b), and hence a paraffin wax volume of 1600 ml was employed. In the experiment series with PVC, the same volume of paraffin wax was cast as a 20 mm thick plate, coated in PVC foil (Fig 2a). The paraffin wax chosen (Tudamelt 40/42, Hansen & Rosenthal KG, Hamburg, Germany) has a relatively low solidification point at 42 °C and a melting point at approximately 40 °C. This is expected to resemble realistic conditions in favoured low-temperature systems [22, 35, 58]. Gas chromatographic analyses by the manufacturer on the paraffin wax quality showed a dense distribution of the chain lengths of around 20 to 23 C-atoms (approx. 80%) within a total range between 17 and 32 C-atoms.

A top cover of the container made of transparent plastic foil (for better visibility not present in Fig 2) minimized evaporation effects. To further shield the experiment from environmental influences and to emulate granular properties of soil surrounding a storage basin in the field, expanded glass granulate (Ecoglas, Steinbach Schaumglas GmbH & Co. KG, Salz, Germany) was used (Fig 2). As a recycling material with a grainsize of 5–8 mm, it is also installed as outer insulation material (thermal conductivity of λ = 0.084 W/mK, [59]) at some existing facilities [27, 36, 60].

For heating the storage media, a laboratory thermostat (Julabo ED immersion thermostat) with an electrical power input of 2 kW was applied (Fig 2c and 2d), whereby the heating coil with a circulation pump was installed in the center of the water column. This simulated a direct loading procedure without thermal stratification in the basin but ensured a homogenous temperature distribution at all interface regions. For temperature measurements and data logging, two 20-channel Keysight 34901A multiplexers and one Keysight 34972A were used. In total, 15 Pt100 temperature probes were connected (stainless steel, waterproof, 4 wires, length 500 mm, measuring tip 20 mm, accuracy 1/10 DIN (German Industry Standard), Fig 2d). The accuracy of the sensors is temperature-dependent. Within the temperatures of all experiments it ranges between ± 0.04 °C (at 20 °C) and ± 0.06 °C (at 60 °C). Three probes were directly cast into the paraffin wax body at different heights. The distribution of the temperature sensors is shown in Fig 1. An HD camera for time-lapse recordings facilitated visual observation.

#### 2.1.2 Testing procedure and data processing

**Fig 3:**
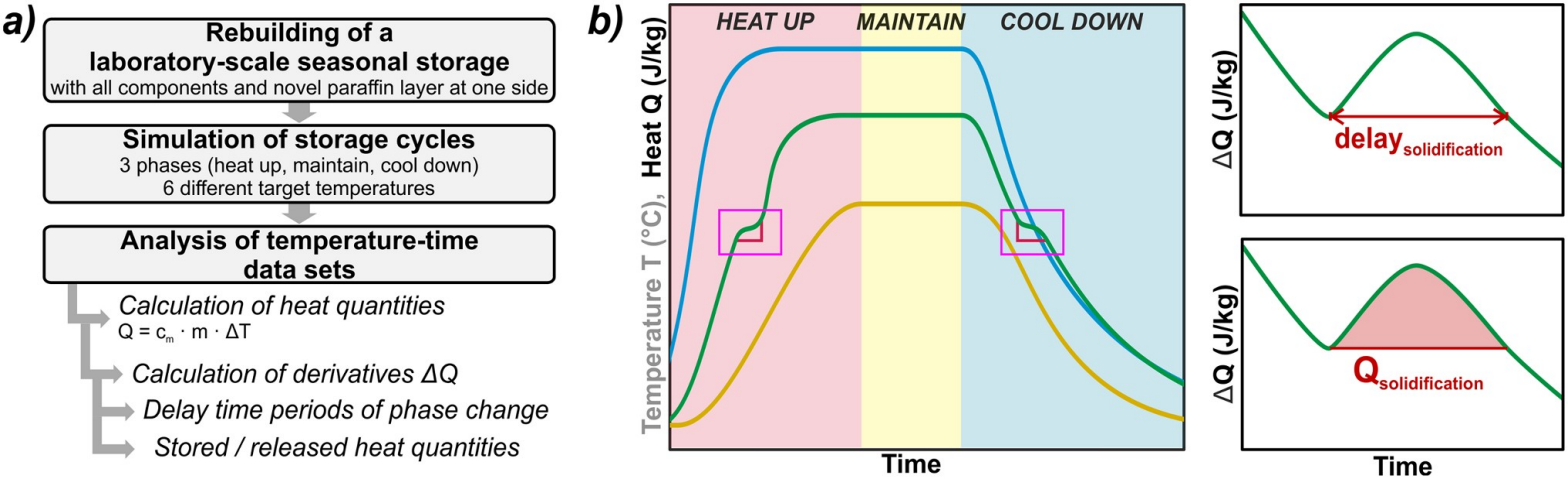
a) Workflow of thermal performance experiments; b) experimental phases with definition of delay and stored heat content (schematic illustration). Pink box: delay in heating/cooling due to phase chance effects. Line colors in Fig 3b: blue: water, green: paraffin wax, yellow: surrounding material.

The overall workflow of the thermal performance experiments is depicted in Fig 3a. After casting the paraffin wax directly into the PS or PVC cavity (Fig 2), all temperature sensors were installed at the respective positions (Fig 1). The experiment was operated in three phases for six different target temperatures between 34 °C and 40 °C. In the first phase, the system initiated at ambient temperature was heated up to the predefined target temperature (heating phase). Although the heating rate could not be directly measured, it was constant for all experiments, because the heating device always operated at full power. The equilibrium state, implying a constant gradient to the environment, was maintained for at least 12 hours (maintaining phase). This second phase was stopped by switching off the heating thermostat and the whole structure cooled down until the ambient temperature was reached again (cooling phase, Fig 3b, left). During all phases, temperatures at all sensor positions were recorded at intervals of 30 s and time-lapse videos (frame rate 100 fps from 30 s image intervals) were recorded with the camera.

For data evaluation, focus was set on two parameters to judge the suitability of paraffin wax for enhancing heat capacity and thermal insulation performance of the membrane: (i) a retardation factor that represents the delay of lateral heat transfer during melting or solidification of the paraffin wax; (ii) the amount of stored heat while melting during the heating phase or, vice versa, recovered from the solidifying paraffin wax in the cooling phase. In the following, all temperature data sets were firstly converted into heat quantities via the deployed masses (*m*) and the specific heat capacities (*c*_*m*_) of paraffin wax, water, and surrounding material, respectively (using the caloric equation for heat *Q* = *c*_*m*_ ∙ *m* ∙ Δ*T*). In this context, the rather heterogeneous quality of the technical paraffin wax was considered by assigning an uncertainty of 5% to the specific heat capacity of the respective material. Combined with the measuring accuracy of the temperature sensors, the prolongated uncertainties of heat values resulted in max. ± 8.5 kJ/kg.

Although phase change effects are already detectable in these datasets (pink box in Fig 3b, left), the derivatives of these curves reveal the changes in the heat content of the paraffin wax more precisely and provide information on the accurate retardation timeframes (Fig 3b, right top). Ultimately, the amount of stored and retrieved heat was quantified using the integral of energy changes over the retardation period (Fig 3b, right bottom).

### 2.2 Leakage tests

#### 2.2.1 Experimental setup

The leakage tests served to prove the desired self-healing mechanism when using paraffin wax in the waterproofing storage membranes. Since it is used in neat form, the material has a direct thermal junction with the interfaces of the inner and outer layers and therefore should first melt in the heating phase. Subsequently, it should be available as a hydrophobic, mobile liquid to clog pathways into the colder surrounding material in case of leakages.

**Fig 4:**
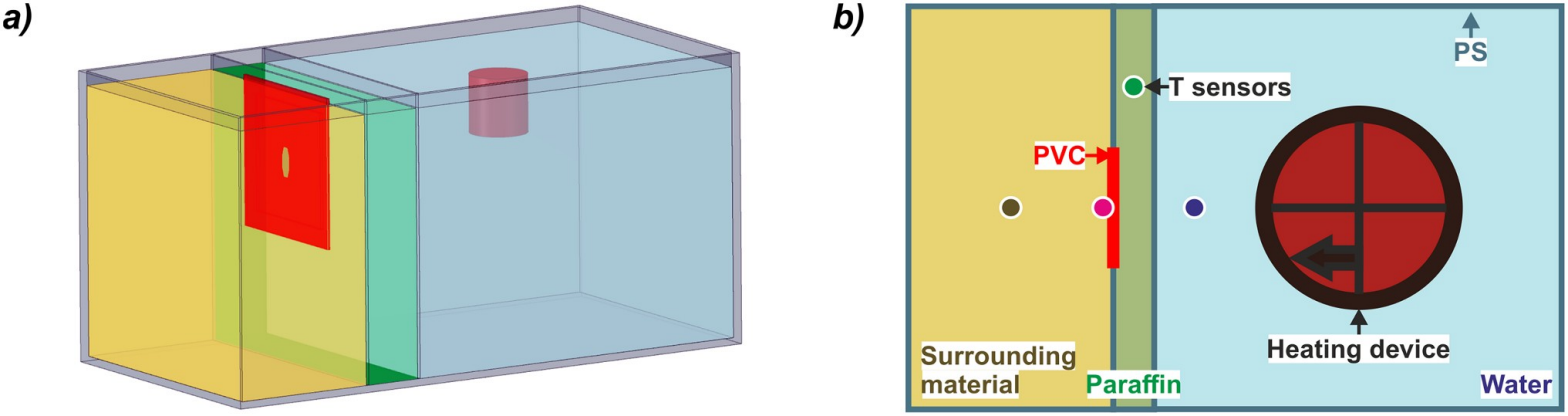
(a) Sketch and (b) top view of leakage experiments. Green: paraffin wax, blue: water, red: PVC layer, yellow: surrounding material. Sensor positions marked with dots. PVC: polyvinylchloride, PS: polystyrene glass plates.

**Fig 5:**
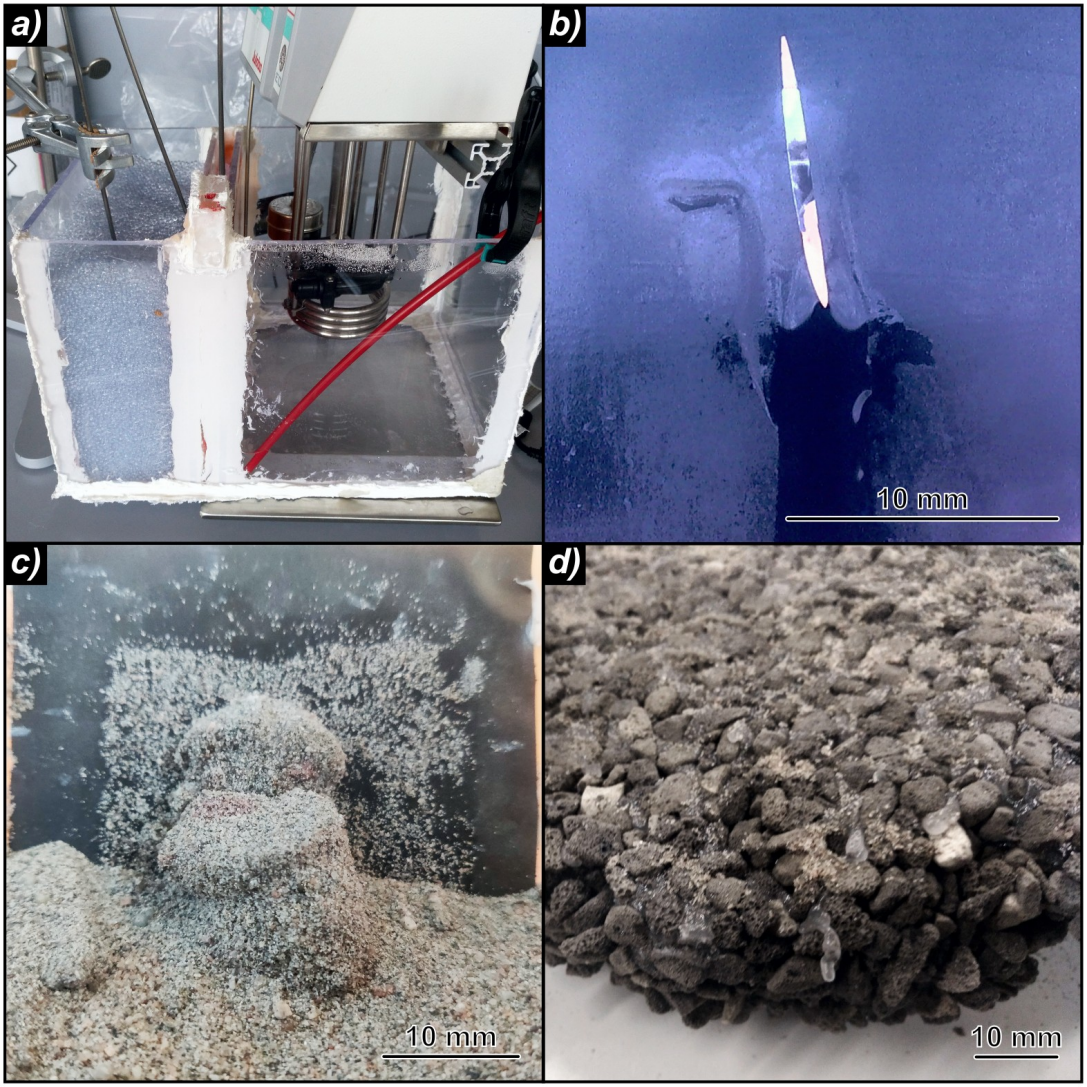
a) Laboratory set up, b) fissure in polyvinylchloride foil with outflowing paraffin wax, c) solid body of sand with paraffin wax, d) impermeable compound of surrounding material with pore spaces filled by paraffin wax.

The set-up is shown in Figs 4 and 5 and, for consistency and comparability, consists of many components of the previous tests. The operation and measurement equipment, such as sensors and the heating thermostat, were the same as in the experiments of thermal enhancements described above (Fig 5). However, a much smaller external PS casing of 400 x 200 x 200 mm (length, width, height) was used to simulate a cross-section through the storage membrane and the surrounding material was only installed on one side (Fig 5a). A 20 mm thick paraffin wax layer (volume: 800 cm^3^) was implemented in direct contact to the interior filling of deionized water (280 mm x 200 mm x 200 mm). In the outer PS plate, a 50 mm x 50 mm wide window was covered with a PVC film to simulate various leakage types in the sealing foil, such as fissures, larger holes and perforated zones (Fig 5b).

The area of the surrounding material finally resulted in a volume of 100 x 200 x 200 mm and allowed to observe and measure the outflow and dispersion behavior of the paraffin wax (Fig 5c and 5b). Two surrounding materials were deployed in separate test series: (i) a fine sand (grain size: 0.063 to 2 mm) was used to reproduce realistic field conditions, while (ii) glass balls with a diameter of 3 mm were chosen to imitate an ideal grain structure and to test the behavior of molten paraffin wax in mediums with a larger pore space (Fig 5a).

#### 2.2.2 Testing procedure and data evaluation

**Fig 6:**
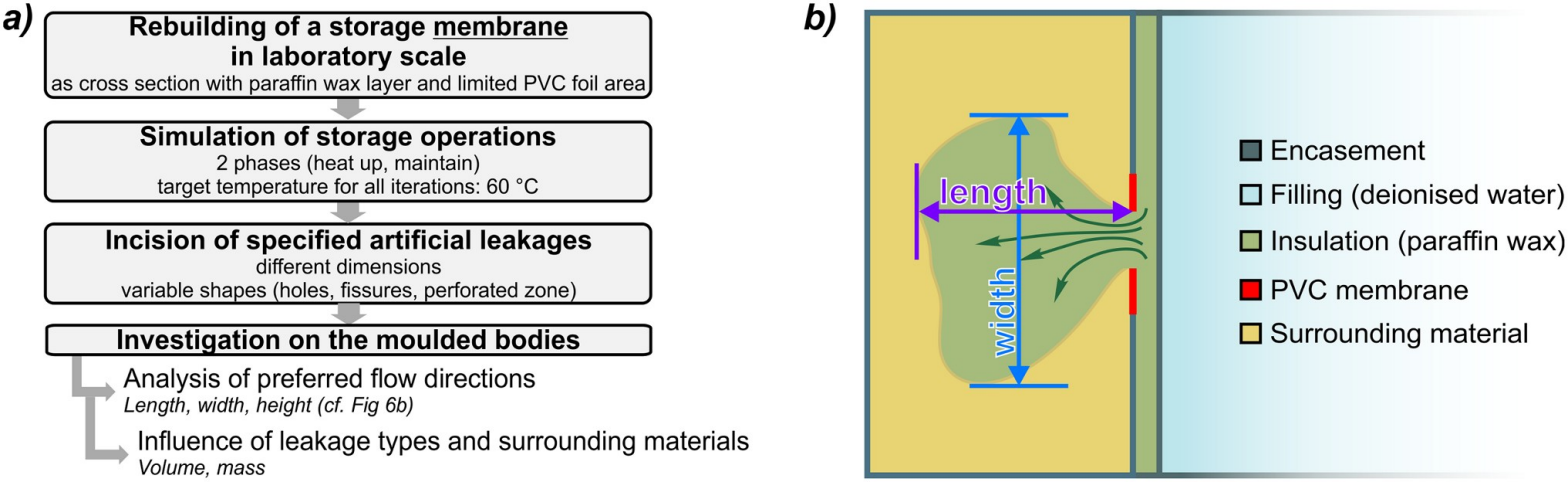
a) Workflow for leakage experiments, b) top view of measurements of formed bodies after induced leakage. PVC: polyvinylchloride.

The workflow of the leakage experiments is shown in Fig 6a. In the different scenarios, a certain type of artificial leakage was incised into the PVC foil and temporarily sealed with adhesive tape. Both vertical and horizontal fissures with an area of 20 mm^2^, a vertical fissure with an area of 40 mm^2^, as well as a large hole with an area of 380 mm^2^, and a foil containing a perforated region (total leakage area of 23.6 mm^2^) were tested. The storage tank was then filled with water and paraffin wax was cast in the cavity of the simulated storage membrane. After the paraffin wax solidified, the adhesive tape was removed, uncovering the defective zone, and the surrounding material (sand or glass balls) was inserted. For a rapid heating, the thermostat was set to 60 °C and the data logging was started. Just as all of the paraffin wax was in liquid state, the thermostat was turned off and the system cooled down to ambient temperature. For evaluation, the compound body of paraffin wax and sand or glass beads embedded in the surrounding material was exposed and sampled. Ultimately, the complete set-up was returned to its initial state for a new iteration.

After each of these tests, the molded compound bodies of solidified paraffin wax and surrounding material were exposed, and their dimensions (length, width, height) and masses were recorded by caliper rule. The volume was calculated via the density and the mass of the compound material. Assuming a measuring precision of 0.5 mm for expansions and 1 g for the weight of the composite bodies, the resulting overall accuracy of the volume data resulted in max. ± 2.7 cm^3^ for bodies of compound material and ± 1.4 cm^3^ for data on volumetric paraffin loss. Special attention was further paid to the observed directions of paraffin wax dispersion. Accordingly, length and width of the bodies were defined as shown in Fig 6b, while the height was measured vertically.

## 3 Results and discussion

### 3.1 Enhancement of thermal performance

#### 3.1.1 Visual observations

**Fig 7:**
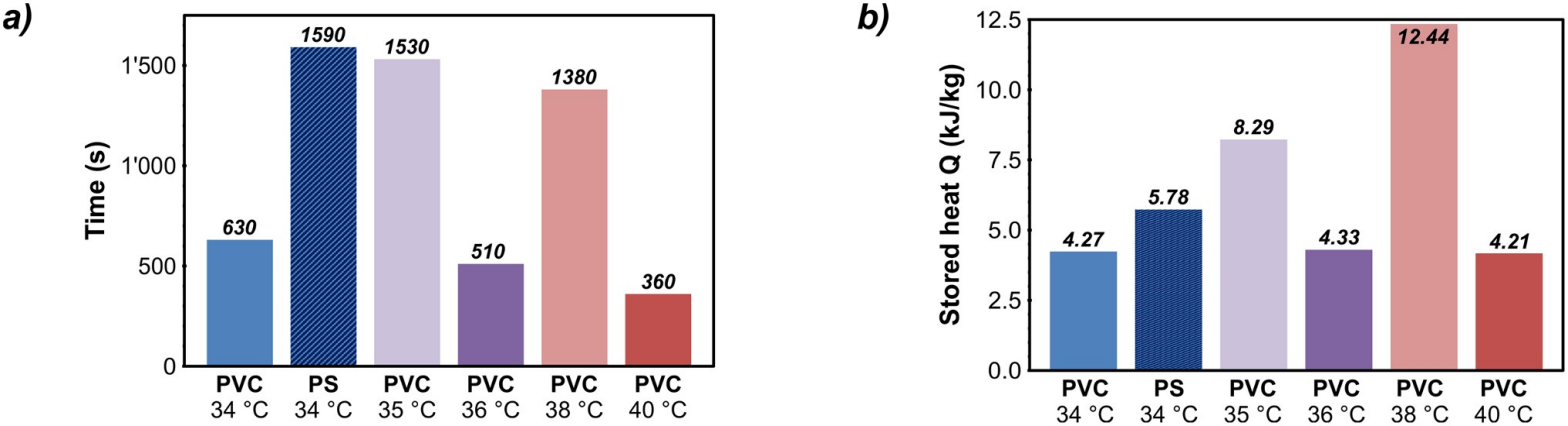
a) Delay in heating of laboratory heat storage due to paraffin wax melting; b) additional stored heat in paraffin wax during heating phase. PVC: polyvinylchloride, PS: polystyrene glass plates.

**Fig 8:**
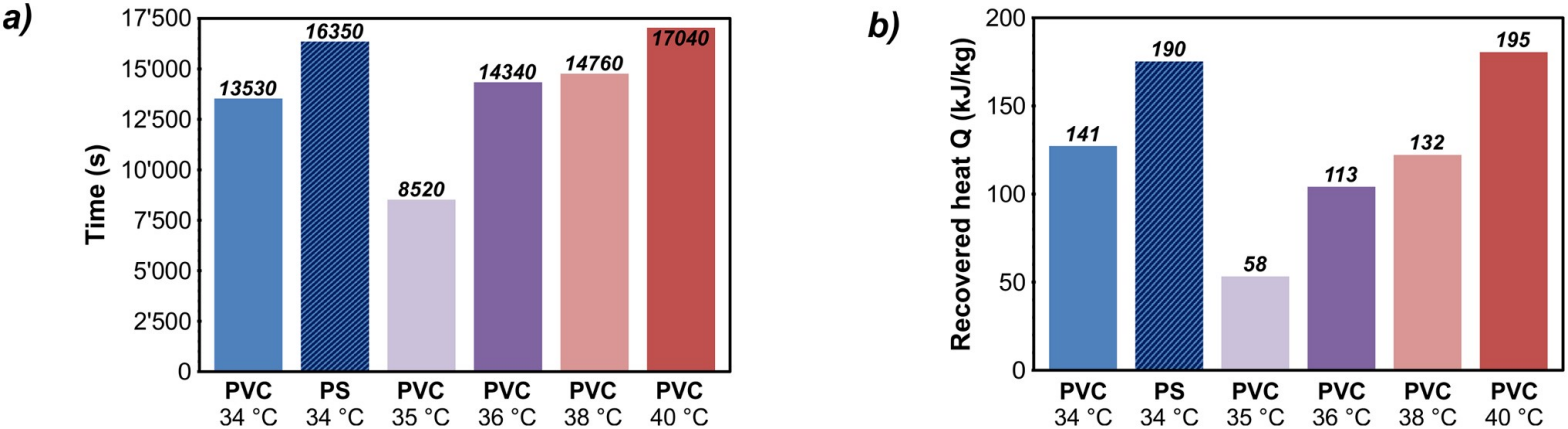
a) Delay in cooling of laboratory heat storage due to paraffin wax solidification; b) additional released heat by paraffin wax measured in the cooling phase. PVC: polyvinylchloride, PS: polystyrene glass plates.

Figs 7 and 8 summarize the results of the thermal performance experiments in both the heating and the cooling phases for six selected experimental settings. Figs 7a and 8a show the retardation by melting or solidification of paraffin wax. In contrast, the absorbed/stored heat in the paraffin wax, which represents the extension of the storage capacity, is depicted in Figs 7b and 8b. The results cover only experiments with PVC as sealing material, except for one iteration with PS for comparison.

First results and striking features of the presented concept are already apparent in the evaluation of the time-lapse recordings, as liquid components could be observed even at low temperatures. Therefore, even experiments with target temperatures below the melting point of the used paraffin wax show significant retardation effects and a storing/recycling of thermal energy. This can be attributed to the paraffin wax composition, as the technical quality employed in the experiments here is not a highly purified material. As it contains different chain lengths of hydrocarbon molecules, fractionalization occurs while heating or cooling and different partial sections melt and solidify in different temperature ranges.

It should be noted that this applies to all induced phase changes, resulting in no clear and sharp but soft and slow transitions. While this can be expected also in applications in practice, this adds uncertainties to the interpretation of the measurements. However, these effects are also taken into account within an uncertainty assessment, since all values of measurements and physical properties were assigned with error coefficients during the data evaluation (see section 2.1.2).

The second result already shown by visual monitoring were deformations of the paraffin wax layer during the melting process when using PVC foils. Displacement of the paraffin wax due to the pressure of the filling in direction of the surrounding material resulted in a wedge-shaped bulge. As a result, the thickness of the paraffin wax layer was substantially reduced at the bottom and enlarged at the top, raising technical questions on robust implementation techniques at the field scale. Even if these deformations could be avoided in the laboratory by using the stable PS plates, for large-scale storage systems it would be problematic to use a configuration between PVC foils without stabilizing structures.

#### 3.1.2 Retardation and energy storage effects in the heating phases

Following the evaluation of visual documentation, the analysis of the temperature data records starts with the heating phase (Fig 7). Thereby the results show significant delays due to the paraffin wax melting in all six test variants. This is remarkable, since this phase is comparatively short with a linear temperature increase of 0.49 to 0.71 K/min.

The range of retardation period values (Fig 7a) among the different experiment settings is high, spanning from 360 s to 1600 s, with an average melting delay of about 1000 s, but a correlation with the applied target temperatures is not apparent. Similarly, due to the given uncertainties, it cannot be deduced from the increased individual value of the test with PS instead of PVC that the use of this alternative material solely generates a higher retardation. Nevertheless, the retardation value of 1590 s for the PS-variant is 80% higher than the average value of about 880 s for the tests with the PVC film. However, the results of all of the test executions prove the desired mechanism to be effective: Based on the retardation times, it can be expected that quick charging of an application storage can be effectively delayed by the melting processes of the paraffin wax. Simultaneously, the results also indicate a reduction of lateral heat losses.

As described in section 2.1.2 and visible in Fig 3b, there is a close correlation between the retardation times and the thermal energy stored in the heating phase (Fig 7b). Therefore, the results of the latter also show large fluctuations, ranging from 4.21 kJ/kg to 12.44 kJ/kg paraffin wax at an average value of 6.55 kJ/kg. While these values are low, it is likely that slower melting processes could not be detected due to the rapid heating. No clear influence of the sealing material could be observed. The difference between PVC and PS at the same temperature is low, and the value for PS of 5.78 kJ/kg is not significantly above the average of 6.71 kJ/kg for all PVC experiments.

In conjunction with the given mass of paraffin wax used (1,200 g for a volume of 0.0016 m^3^ or 1.6 l and a density of 750 kg/m^3^), the results of stored energy quantities are used for a linear upscaling to field conditions. Based on the frustum geometry of common pit thermal energy storage systems (PTES) [24, 29, 31], a storage volume of 50,000 m^3^ and a thickness of the paraffin wax layer of 0.1 m, a paraffin wax volume of 1000 m^3^ can be assumed.

The results ultimately reveal an enhancement of storage capacity for this application case of about 3.16 ∙10^6^ MJ or 0.88 MWh to 9.33 ∙10^6^ MJ or 2.59 MWh. This additional energy reservoir would thus be available during a quick and intense charging process by applying the paraffin wax. However, compared to the storage capacity of the PTES water filling of 1.16 GWh (for a temperature spread of 20 K and a storage capacity of water of 4.19 kJ/kgK), this is a small benefit and thus the additional heat storage capacity only is not sufficient for justifying the use of paraffin wax.

#### 3.1.3 Retardation and energy storage effects in the cooling phases

For comparison, the same measurement series was considered for evaluation of the cooling phases (Fig 8). As expected from Fourier’s law [61], the cooling phase is not reflected by a linear gradient of temperature and energy content, but by an exponential decrease converging to the ambient temperature. As a result, this phase covers much longer time frames until the ambient temperatures are reached again (Fig 8a, average 95 h, max. 144 h).

The first results of the cooling phase already show substantial differences as the retardation periods caused by the solidification of the paraffin wax are several orders of magnitude higher (Fig 8a). They range between 8,500 s (~ 2.5 h) to about 17,000 s (~ 4.7 h), with an average value of 14,000 s (~ 3.9 h). Furthermore, a remarkable difference between the values for PS and PVC at the same temperature (34 °C) indicates a significant influence of the sealing material, since more paraffin wax can be utilized when deformation processes are prevented. However, there is no distinct trend observable for longer retardation times at higher operating temperatures. Altogether, results of retardations in the cooling phase demonstrate a more efficient applicability of the presented approach. Due to the long-lasting delays, subsequent energy can be provided in the marginal area of the storage in case of a rapid discharge of a storage unit. As a result, the steepness of thermal gradients in towards the surroundings can be reduced and energy losses are minimized.

By evaluating the cooling phase, results on energy recovery can also be determined more accurately (Fig 8b). This is because this experiment phase represents an unaffected cooling process in contrast to the heating phase, where rapid energy inputs to the filling may superimpose phase change effects within the paraffin wax. As a consequence, the cooling phase allows to observe the entire sequence of phase change effects without interferences of external energy flows, also enabling much slower processes to be resolved. Hence, the results of energy recovered from the paraffin wax are several orders of magnitude higher than those determined during heating with an average of 138 kJ/kg at a range from 57 kJ/kg to 195 kJ/kg. Although a natural cooling curve as applied in the experiments does not properly represent the conditions of intermittent storage discharging in an application case, the findings prove that cooling is delayed by the energy recovered from the paraffin wax solidification. Thus, short-term discharge processes could be buffered and compensated over a longer period, resulting in slower temperature decreases in the storage shell and therefore in less impact on the sealing material’s durability.

The amount of recovered heat is not affected by the operating temperature, but by the sealing material. The more stable PS construction with a constant and uniformly interface ensures the utilization of a larger volume of the paraffin wax. The recovered energy of 190 kJ/kg is significantly higher than the corresponding value using PVC (141 kJ/kg) and it is even higher than the average of all measurements using PVC (138 kJ/kg).

The values of the energy recoveries of the cooling phases are also applicable for scaling to the previously described use case scenario of a 50,000 m^3^ PTES. At this, the results show a striking difference: The volume of 1,000 m^3^ paraffin wax would provide an additional storage capacity of 12.01 MWh to 40.70 MWh (average: 28.77 MWh), being additionally available during a slow cooling or discharging process. Ultimately, these results are by one order of magnitude higher than those of the heating phases. They show an effective utilization of the desired processes and an added value of the new concept in terms of thermal enhancement of seasonal heat storage systems.

### 3.2 Leakage mitigation

#### 3.2.1 Dispersion directions

**Fig 9:**
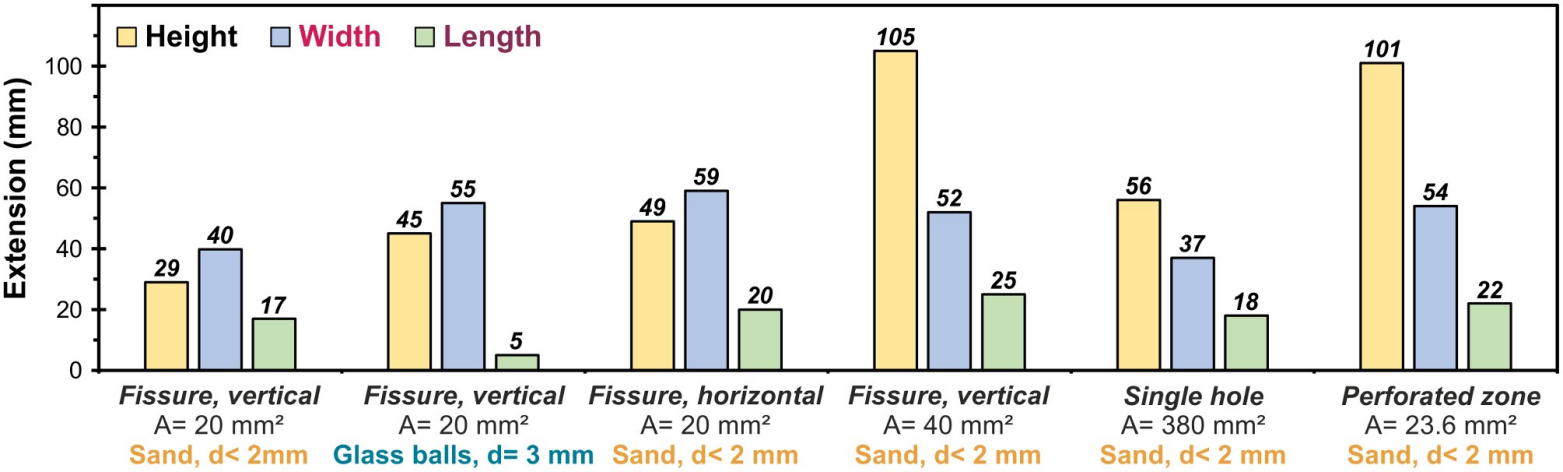
Measurements of formed bodies after paraffin wax loss. The lengths and widths were defined as depicted in Fig 7b, while the height of the solids was measured vertically. A: total surface area of passageways as size of the leakage, d: grainsize of the surrounding material.

The self-healing properties of the newly presented concept are based on the desired mechanism of actively sealing leakage pathways by the hydrophobic paraffin wax. Within this second series of tests, six different scenarios were examined. Fig 9 shows the dimensions of the molded bodies of paraffin wax and surrounding material (according to Fig 6b) in relation to the respective type of leakage and the surrounding material. In one scenario, glass balls were used as surrounding material instead of sand. As the shapes of the different types of leakage (fissures, circular shaped apertures and perforated zone) are very different, it is not expedient to consider their lengths or diameters. Instead, the total surface area of these passageways is used as an auxiliary parameter for comparing the size (“A” in Fig 9) of the leakage.

For all leakages, the results show a dominating dispersion in vertical direction (height) and horizontal to the surface of the sealing membrane (width). The heights, which show the greatest variance (29 mm to 105 mm), indicate a trend with increasing surface area of the leakage. These also comprise the smallest values, being between two and nine times lower compared to the vertical extents. In contrast, the values of widths and lengths scatter around their averages of 49.5 mm and 17.8 mm, respectively, within small ranges (min: 37.0 mm and 5.0 mm, max: 59.0 mm and 25.0 mm).

These results can be attributed to the influence of the gravitational force. Thereby the paraffin wax preferentially flows along the outer PVC foil of the storage membrane and spreads mainly vertically. However, the horizontal spread along the sidewall of the storage is also remarkable, which even exceeds the vertical component in case of smaller fissures. The high temperature at the outer membrane interface obviously plays a crucial role, ensuring that the paraffin wax does not immediately solidify after flowing out of the insulation layer. Thus, it can propagate laterally with significant material losses, representing a major weakness of the suggested overall concept.

Nevertheless, the self-healing mechanism of the presented concept already proves to be effective within these results, since dispersal in a horizontal direction straight away from the storage is successfully hindered. In this regard, the cold surrounding material represents an effective barrier, leading to a rapid solidification of the paraffin wax and clogging of the leakage.

#### 3.2.2 Influence of leakage types and surrounding materials

The evaluation regarding the influence of the type of leakage (hole, fissure or perforation) does not initially reveal any major disparities. However, there are indications that vertical fissures of the same surface area uniformly lead to smaller spreadings of the paraffin wax in all directions. Additionally, in the case of fissures, larger defects enhance propagations in vertical direction.

A more striking difference becomes apparent when comparing the two surrounding materials (glass balls and sand): Even though the pore space in the larger and uniform glass balls provides significantly more volume for dispersal, the length is reduced while the expansion in width and height is increased. Here, the glass bead provides the advantage of a larger reservoir of cold, preventing a flow into the surrounding material and allowing for a faster solidification but causing a deviation along the other two directions.

**Fig 10:**
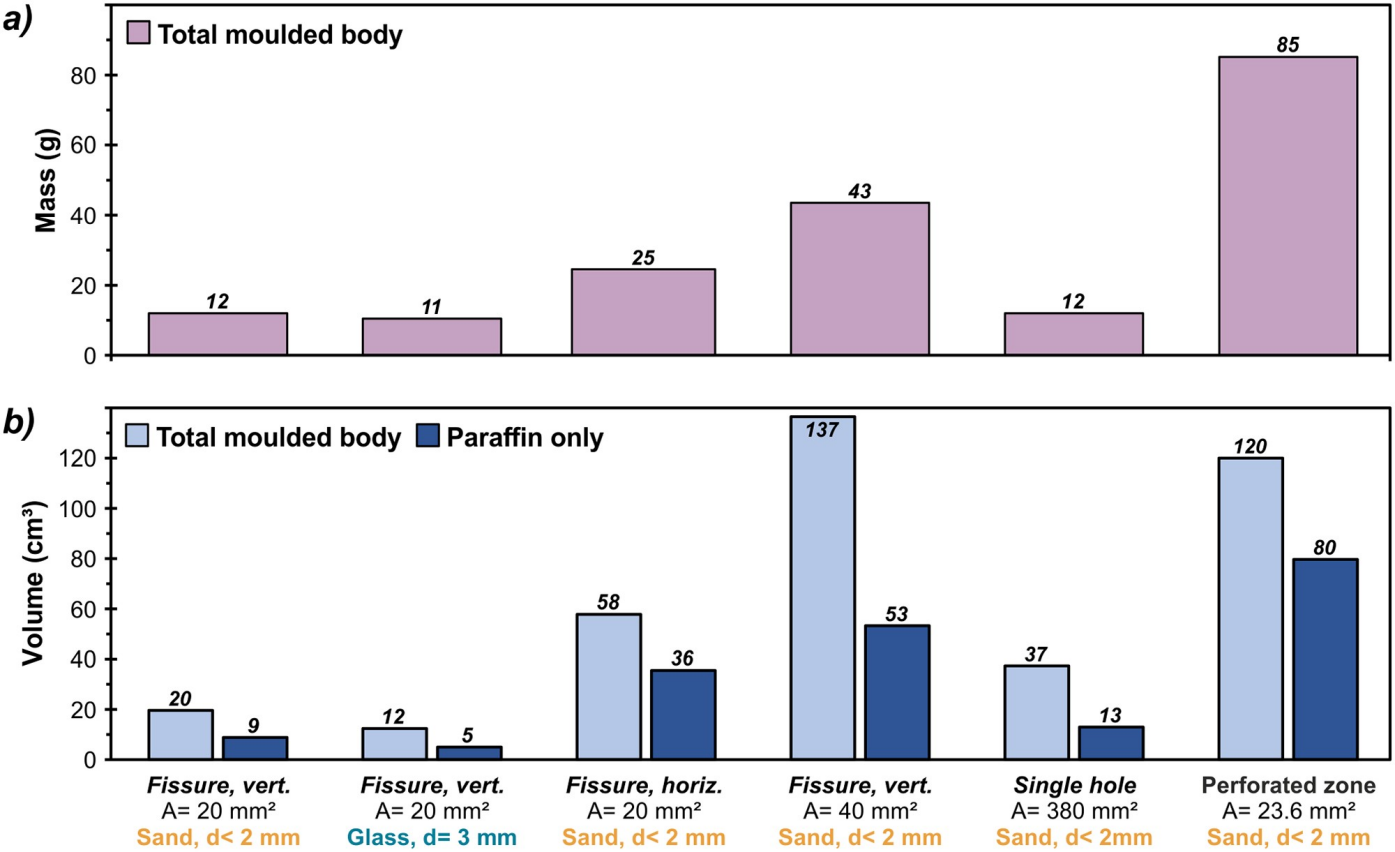
Masses (a) and volumes (b) of formed bodies consisting of paraffin wax and surrounding material, after induced leakage. vert: vertical fissure, horiz: horizontal fissure, A: total surface area of passageways as size of the leakage, d: grainsize of the surrounding material.

Further parameters for the analysis of leakage types and surrounding materials include the mass of the molded bodies as well as their bulk volumes and finally the volume of the dispensed paraffin wax (Fig 10). For comparison, the same series of experiments as in the previous analysis of dispersion directions are presented. The masses of the formed bodies (Fig 10a) show a comparatively small range with values from 11 g to 85 g. The maximum mass value resulted from the experiment of the perforated PVC foil, with its value of 85 g being almost three times higher than the average paraffin wax loss of 31.3 g. A difference in the surrounding material is not evident in these results, but there is again a noticeable difference in horizontal instead of vertical fissures. For the horizontal fissure, the value is more than doubled from 12 g to 25 g.

The results of total volumes (Fig 10b) reveal more distinct differences between the individual configurations. As already indicated in the previous evaluation of the bodies’ dimensions, dispersions also vary in their volumetric extent, ranging from 12 cm^3^ to 137 cm^3^. The share of paraffin wax in the compound bodies ranges from 36% to 67%, implying an additional variability in the volumes of released paraffin wax. Therefore, these data contain a comparatively larger span of 5 cm^3^ to 80 cm^3^. Related to the total volume of 800 cm^3^, paraffin wax losses are small, ranging from 1.5% to 17%. These results prove that the self-healing properties can be applied without major discharges of the material used and that the proposed approach works effectively.

The surface area of the defectives revealed as the most significant influence on the amount of paraffin wax lost, whereby elongated fissures represent a more severe problem than a circular, locally limited hole with a 10 times larger surface area. These results discovered by the tests can be explained by the optimal relationship of circumference to surface area of a circle. Conversely, linear leakages, e.g. like fissures, embody a larger heat reservoir, resulting in a wider surrounding warm surface which favors lateral propagation. The same applies to a perforated and porous foil, which, for example due to material fatigue, has a large number of small defectives. Moreover, a comparison of vertical versus horizontal fissures of the same size and surface area indicates a greater loss of paraffin wax for horizontal gaps in the outer sealing foil. Here, in comparison to vertical fractures, the vertical forces affecting the storage shell intensify the divergence of the incision gap.

Regarding the different types of leakage, the results ultimately show that there are distinct influences of both, the shape, size and orientation of the various defects. Nevertheless, although there are many different drivers, it is proven for all test series that the use of paraffin wax not only adds thermal benefits for seasonal heat storage systems, but can also provide longer operating times by eliminating the problems of material fatigue due to the new inserted self-healing feature.

## 4 Conclusions

The seasonal storage of thermal energy in large-scale basins already offers high potential to increase the flexibility of district heating networks by balancing out fluctuating regenerative energy sources. However, many of the present systems show deficiencies in regard to their thermal performance (emerging as excessive energy losses, significantly reducing profitability) and in regard to their technical set-up, as leakages in sealing foils can result in total system failures. This study tackles these key aspects by introducing a radically new concept for a combined insulation and sealing membrane. Starting as an unconventional approach, the use of paraffin wax as a hydrophobic and latent heat storage material in the marginal area of the storage was extensively evaluated in the laboratory and tested in two separate experiment series.

The advantages of the concept in regard to thermal optimization of seasonal storages were proven by the following results:

- A fast availability of these processes during a rapid heating of the storage filling was observed after only a few minutes.
- Conversely, a uniform energy recovery from the paraffin wax was observed during natural cool-down over periods of 2.5 to 4 hours.
- The additionally usable amount of heat provided by the paraffin wax revealed values in the laboratory of around 6.55 kJ/kg during the intensive heating phase and around 138 kJ/kg during the slow cooling phase.
- For full-scale application cases, a theoretical scale-up indicated a storage capacity expansion of up to 40.70 MWh.

One the one hand, these results show that both the buffering of intensive, short-term charges and discharges as well as normal operation can be optimized as desired. In this respect, the advantages of a low-cost storage material with a fast thermal applicability (water) and a latent, more stolid storage material (paraffin wax) are perfectly combined for maximizing long-term performances by increasing total storage capacities and reducing thermal stress on the materials because thermal gradients are flattened.

On the other hand, certain disadvantages of the new concepts in regard to its thermal behavior have to be mentioned:

- The added storage capacity by the paraffin wax represents only a small fraction of the total storage capacity, raising questions regarding the economic viability.
- A technical issue was detected, as the previously uniform layer of paraffin wax was deformed into a wedge-shaped structure, leaving no paraffin wax remaining in the lower section.

Although this technical disadvantage could be diminished by the incorporation of thermal stratification, further technical refinements are advisable, involving for example the application of a suitable support structure. However, attention has to be paid to the appropriate relationship between investment and added value in further developments of the concept, which would not be satisfactory considering only the total capacity increase.

With this foresight, the second series of experiments investigated the technical feasibility of self-sealing effects and also revealed significant benefits of the newly presented approach:

- After emerging from artificially incised leakages, the paraffin wax cooled down already after very short distances from the sealing foil.
- Furthermore, the proportions of lost paraffin wax are comparatively small between only 1.5% to 17%.
- Especially with coarser-grained substrates, the molded bodies led to the intended clogging of defects in the sealing membrane.
- Thereby, the mechanism was effective for all scenarios of different leakage types and sizes of the defects as well as in case of different surrounding materials.

However, by analyzing the several influencing parameters, it was also possible to identify potential shortcomings and technical disadvantages of the presented concept:

- Especially in the case of widely distributed, diffuse material deficiencies (e.g. perforations), an increased risk of large paraffin losses prevails because heat can be continuously supplied over a large area by the remaining paraffin layer.
- The warm surface of the storage shell allowed the paraffin wax to spread along this structure in the direction of the gravitational force.
- With these preferred flow directions, a total closure of the leakage might not be ensured because the desired clogging effect can only be realized in one direction, while dispersion along the outer storage surface can still lead to major losses of paraffin wax.

In conclusion, the general objective of the study as a proof of concept is successful, although unexpected risks are for now hindering a direct implementation to large-scale application cases. Both thermal utilization and an enhancement of the storage capacity could be demonstrated, but the paraffin wax proved to be highly mobile after the melting process, which also poses a problem to the desired self-sealing mechanism. Consequently, future attention will have to focus exclusively on technical aspects in order to guide this promising concept to field-scale implementation.

## Supporting information

S1 Table Temperature measurement records of the experiments on the enhancement of thermal performance (cf. sections 2.1 and 3.1). (XLSX)

S2 Table Records of the experiments on leakage mitigation (cf. sections 2.2 and 3.2). (XLSX)
